# A Rapid Magnetofluidic Micromixer Using Diluted Ferrofluid

**DOI:** 10.3390/mi8020037

**Published:** 2017-01-25

**Authors:** Majid Hejazian, Nam-Trung Nguyen

**Affiliations:** Queensland Micro and Nanotechnology Centre, Griffith University, Brisbane, QLD 4111, Australia; majid.hejazian@griffithuni.edu.au

**Keywords:** magnetic, microfluidics, mixing, magnetoconvection, ferrofluid

## Abstract

Effective and rapid mixing is essential for various chemical and biological assays. The present work describes a simple and low-cost micromixer based on magnetofluidic actuation. The device takes advantage of magnetoconvective secondary flow, a bulk flow induced by an external magnetic field, for mixing. A superparamagnetic stream of diluted ferrofluid and a non-magnetic stream are introduced to a straight microchannel. A permanent magnet placed next to the microchannel induced a non-uniform magnetic field. The magnetic field gradient and the mismatch in magnetic susceptibility between the two streams create a body force, which leads to rapid and efficient mixing. The micromixer reported here could achieve a high throughput and a high mixing efficiency of 88% in a relatively short microchannel.

## 1. Introduction

Rapid mixing in microfluidic devices is an important task for material synthesis, chemical/biochemical analysis, and cooling [1]. Micromixers have a broad range of applications such as for reactors [2,3,4], lab on a chip for chemical engineering [5], enhancement of chemical selectivity [6], extraction processes [7], drug discovery [8], polymer synthesis [9], and DNA amplification [10].

The challenge in designing micromixers is to achieve fast and efficient mixing within a short residence time or in a short microchannel. Depending on their operation mechanism, micromixers are categorized as passive or active. Passive mixers are easy to fabricate due to the fact that they do not need an external energy supply. Passive micromixers include lamination [11,12], chaotic advection, injection, or droplet mixers [13]. Faster and more effective mixing can be achieved using active micromixers, which require an external energy source and a more complex fabrication process. For stirring or disturbing the fluid flow, active concepts such as acoustic [14], dielectrophoretic, electrokinetic, pressure perturbation, electro-hydrodynamic, magnetic, and thermal actuations have been used in active micromixers. A number of excellent review papers were devoted to the classification and application of micromixers [15,16,17,18,19].

Fluid flow in a microfluidic device can be manipulated using magnetic forces [20]. Inducing a magnetic force is wireless and has the advantage of providing an environment for cell viability in biological studies [21]. Several methods have been described which use magnetic forces for mixing on a microscale: micro magnetic stirrers, magnetophoresis of magnetic particles, magnetohydrodynamics (MHD), and micro magnetofluidics [22]. Chaotic mixing of magnetic beads in a biological fluid was achieved using induced magnetophoresis [23]. The micromixer consists of micro conductors embedded in a microchannel. The chaotic regime was verified by the numerical analysis of particle trajectories. The driving frequency and the residence time could be adjusted to obtain effective mixing. Zolgharni et al. investigated chaotic mixing of magnetic particles in a biological fluid using the Lagrangian tracking method [24]. The efficiency of capturing the target cells with magnetic particles was evaluated. Numerical simulation allows for the optimization of the operating conditions for the mixer. Bau et al. reported theoretical and experimental studies on a MHD-based micromixer [25]. An array of electrodes in the presence of a magnetic field induces a body force in the fluid, which generates a complex flow field. The concept deforms and stretches fluid interfaces to enhance mixing.

Rida and Gijs introduced a novel method for mixing based on the manipulation of self-assembled magnetic microbead structures held in place by a magnetic field [26]. Soft ferromagnetic structures were integrated in a Y-shaped microchannel to create local magnetic fields. Efficient mixing could be achieved as a result of the strong particle-liquid interaction. A mixing efficiency of 95% within a length of 400 µm was achieved. Adjusting the magnetic field and the liquid flow rate optimizes the mixing process. Wang et al. numerically investigated a magnetic particle driven micromixer [27]. The effect of parameters such as magnetic actuation force, switching frequency, and dimensions of the microchannels were studied. Based on the numerical results, the maximum efficiency occurs at a relatively high operating frequency for large magnetic actuation forces and a narrow microchannel.

With manipulation of an entire ferrofluid stream using a magnetic field, Mao and Koser achieved rapid mixing in a microchannel using magnetofluidic actuation [28]. Embedded electrodes carrying traveling magnetic waves were used to create a local magnetic field. Mixing of water-based ferrofluids with a fluorescein buffer solution demonstrates a significant enhancement in mass transport as compared with that of pure molecular diffusion. An alternate-current (AC) electromagnetic field can induce transient flows between a ferrofluid and a fluorescence dye solution, allowing for a simple and efficient mixing concept [29]. The magnetic field causes significant and uniform expansion of the ferrofluid toward the dye, creating extremely fine fingering structures at the interface. High mixing efficiency of up to 95% was achieved within 2 s and at a distance of 3 mm from the inlet of the microchannel. Numerical simulation of the phenomenon was reported in a separate work [30]. The results from the simulation demonstrate that the magnetic body force significantly affects mass transport of the ferrofluid.

Zhu and Nguyen reported the mixing phenomenon and the effect of a uniform magnetic field on a superparamagnetic ferrofluid [31]. A mixture of deionized (DI) water, glycerol and water-based ferrofluid were introduced into a circular microchamber. As a result of magnetic susceptibility mismatch under a uniform magnetic field, instability at the interface was observed leading to rapid mixing. The effect of parameters such as magnetic flux density, flow rate ratio, and viscosity ratio on the mixing efficiency was investigated. Kitenbergs et al. examined the mixing process of a superparamagnetic ferrofluid stream with and the diamagnetic water stream under a homogeneous magnetic field in a Hele-Shaw cell [32]. Mixing enhancement through magnetoconvective transport was demonstrated.

Using fluorescent dye, the extent of mass transport through magnetofluidic phenomena can be investigated [33]. We demonstrated in our previous work [34] the expansion of a ferrofluid stream containing non-magnetic particles under the effect of the magnetic body force created by a uniform magnetic field. The difference in magnetic susceptibility created a strong enough body force to carry along the diamagnetic micro particles in the same direction as the magnetic nanoparticles. We further demonstrated that without a susceptibility mismatch between the streams and only utilizing a nonuniform magnetic field, diamagnetic microparticles in a ferrofluid can be deflected [35]. Recently, we utilized a simple hydrodynamic focusing system to investigate mixing of ferrofluid and water [36]. The core stream was ferrofluid mixed with diamagnetic fluorescent dye. We demonstrated the spreading of the core stream under a non-uniform field of permanent magnets. Compared to passive diffusion, significant improvement in mass transfer was observed.

In the present paper, we propose a simple, efficient, and low-cost micromixer. We examined the mixing of a ferrofluid stream into a water stream in a non-uniform magnetic field at different flow rates and concentrations. Furthermore, we evaluated the mixing efficiency using the recorded images of a fluorescent dye. This simple device has the advantage of exploiting both susceptibility mismatch and non-uniform magnetic fields, resulting in a strong mixing effect. On the other hand, using low-cost permanent magnets allows for the simple design and fabrication of the device. Our mixer could be integrated as a part of a lab on a chip system for chemical or biological applications.

## 2. Materials and Methods

**Figure 1:**
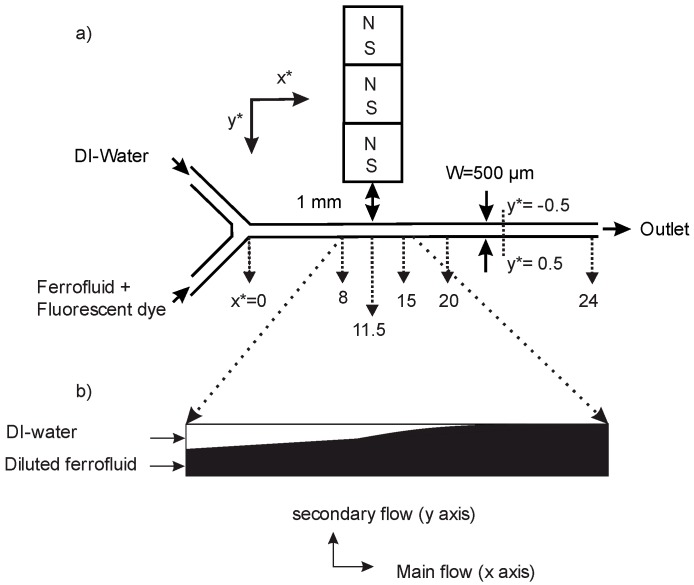
Schematic of the experiment (**a**) Experimental setup (x* = *x*/*W*, y* = *y*/*W*); (**b**) Deflection of superparamagnetic ferrofluid stream towards the magnets due to magnetoconvection, based on experimental observation.

Figure 1a depicts a schematic of the micromixer. The micromixer is a straight rectangular channel with two inlets and one outlet. The microchannel has a depth of *H* = 50 µm, a width of *W* = 500 µm, and a length of *L* = 12 mm. The device was fabricated out of polydimethylsiloxane (PDMS) using the standard soft lithography technique, which was described briefly in our previous work [36]. Two precision syringe pumps (SPM100, SIMTech Microfluidics Foundry, Singapore) were used to feed the fluids into the microchannel. The microfluidic device was placed on an inverted microscope (Nikon Eclipse TS 100, Nikon Corporation, Tokyo, Japan) equipped with a high-speed camera (Photron 120K-M2, Photron, Tokyo, Japan) for visualization. Deionized (DI) water was fed into the microchannel as the diamagnetic stream. The commercial water based ferrofluid (EMG707, Ferrotec, Santa Clara, CA, USA), containing 2% volume concentration (ϕ = 2% vol.) magnetic nanoparticles, was used to make a diluted superparamagnetic solution for the second stream. Nominal nanoparticle diameter was 10 nm. Initial magnetic susceptibility of the commercial ferrofluid was 1.51 (International System of Units (SI)). For visualization purposes, 0.05 g of Fluorescein sodium salt (acid yellow, Sigma-Aldrich Co., St. Louis, MI, USA) was dissolved in 20 mL of DI water. The commercial ferrofluid was then diluted with the fluorescent dye solution to make ϕ = 2% and ϕ = 20% vol. ferrofluid concentrations. Three identical 3.2 mm^3^ neodymium–iron–boron (NdFeB) permanent magnets (B222, K&J Magnetics Inc., Pipersville, PA, USA) were used to create the non-uniform magnetic field for the mixing experiments. A slot was cut in the PDMS to embed the magnets into the device. The position of this slot was approximately in the middle of the microfluidic channel. For this experiment, the magnets were positioned in a way that allowed us to observe the following: the secondary flow, the magnetic stream approaching the upper wall, and mixing of the streams before and after the magnets. The experiments were carried out with the constant flow ratio of 1, for a wide range of flow rates from 2 to 300 µL/min and two concentrations of the ferrofluid (2, 20% vol.). The magnetic field of the permanent magnets was measured as a function of distance and calibrated using a Gauss meter (Hirst Magnetic Instruments Ltd., Falmouth, UK) and reported in our previous work [36]. The distance between the magnets to the channel wall was 1 mm. Across the microchannel width, the magnetic flux density drops from approximately 250 mT to 175 mT.

## 3. Results and Discussion

The micromixer reported here takes advantage of the non-uniform magnetic field provided by permanent magnets placed next to the microchannel. Six images were taken at different locations along the channel: *x* = 0, 4, 5.75, 7.5, 10, and 12 mm. The magnets were located between *x* = 4 mm to *x* = 7.5 mm (Figure 1a). We normalised the length scale by the channel width *W* (x* = *x*/*W*, y* = *y*/*W*). The distance between the magnets to the upper wall of the channel (y* = −0.5), and the ratio between flow rates of the two streams was held constant for all experiments. In order to investigate the effect of magnetic susceptibility on mixing efficiency, two diluted ferrofluid samples with the concentration of 2% and 20% vol. of the commercial solution were prepared. The stream next to the magnets was DI-water. Mixing of the two streams is only possible if the magnetic solution is introduced through the lower channel. This configuration is best to demonstrate the secondary flow perpendicular to the direction of main flow (Figure 1b) caused by the susceptibility mismatch between the streams. The magnetic stream travels half of the channel width to approach the permanent magnets.

We hypothesise that magnetic susceptibility difference between the two streams creates a magnetoconvective secondary flow [36,37] in the direction of the maximum of the magnetic field. Our aim is to take advantage of the strong, secondary, magnetoconvective flow to achieve efficient mixing of the two streams. For this purpose, different flow rates were tested for each concentration to observe the effect on mixing efficiency. In addition, an extra experiment on testing a wide range of flow rates while holding other parameters constant revealed the optimum operating conditions for our mixer. Diluted ferrofluid was mixed with fluorescent dye for tracing the ferrofluid stream and for evaluating mixing efficiency. The images from the experiment were taken a few minutes after changing the flow rate to make sure that the system has reached a steady state condition.

### 3.1. Fluorescent Signal and Qualitative Analysis

We analysed the fluorescent intensity *I* to investigate the effect of flow rate and concentration on the quality of mixing and to demonstrate the phenomenon. The strength of the fluorescent signal is representative for the concentration of the dye. For this purpose, we use normalized intensity [33]:

(1) $$I^{*}=\frac{I-I_{\min}}{I_{\max}-I_{\min}}$$

**Figure 2:**
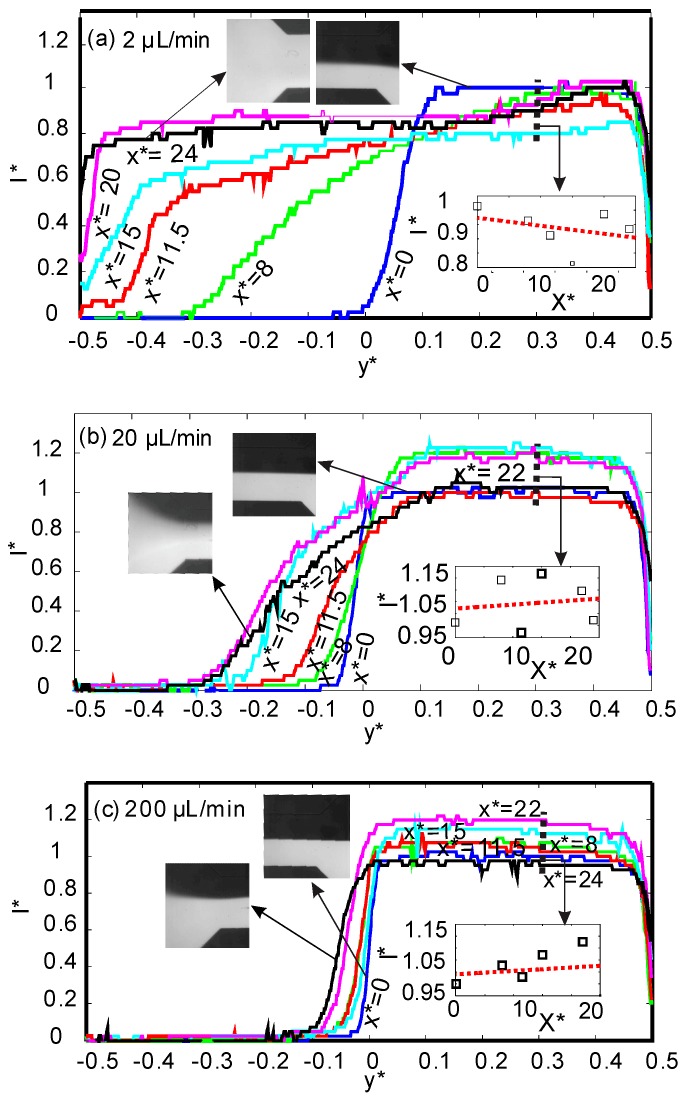
Normalized intensity across the normalised channel width y* for six positions of x* along the mixing channel with a ferrofluid concentration of 2% vol. (x* = 0: blue, x* = 8: green, x* = 11.5: red, x* = 15: cyan, x* = 22: purple, x* = 24: black) and three flow different flow rates: (**a**) 2 µL/min; (**b**) 20 µL/min; (**c**) 200 µL/min.

The measured dimensionless intensity is assumed to be the same as the normalized concentration of the dye molecules c* ($I^{*}=c^{*}$). *I*_max_ and *I*_min_ were measured at the inlet (x* = 0) where the intensity distribution of the fluorescent dye is not affected by the magnetic field. The measurement of the normalized intensity is made on a line across the channel width. The fluorescent signal is evaluated at six positions along the channel’s length (x* = 0, 8, 11.5, 15, 20, 24). Figure 2 shows the normalised intensity distribution of all positions along the y* axis for three total flow rates of 2, 20, and 200 µL/min and for the lower ferrofluid concentration of ϕ = 2% vol. Superparamagnetic iron oxide nanoparticles deflect towards the magnets and create a mismatch in magnetic susceptibility and a secondary flow. As a result, the non-magnetic dye molecules are transported and follow the same path of the nanoparticles. Therefore, the mixing efficiency can be evaluated by analysing the fluorescent signal.

Figure 2 indicates that for all flow rates, the superparamagnetic stream at the inlet (x* = 0) was not affected by the magnetic field, and the intensity profile suddenly drops from I* = 1 to I* = 0 at the interface of the two streams. The effect of the magnetic field on the intensity distribution becomes apparent as the fluid flow approaches the magnets at x* > 0. For the lowest flow rate of 2 µL/min, the intensity profile becomes flat, Figure 2a. A flat intensity profile means a uniform concentration distribution across the width of the channel and complete mixing. As the flow rate increases to 20 µL/min, the hydrodynamic force acting in the flow direction becomes dominant and prevents mixing. As a consequence, the deflected superparamagnetic stream cannot reach the upper wall (y* = −0.5). This effect is more apparent at the higher flow rate of 200 µL/min, where the effect of the magnetic field and mixing are almost negligible.

Another interesting phenomenon that can be observed from the intensity profiles is that the relative intensity values may exceed the value of 1 for the value of y* > 0. The higher intensity values are the result of magnetophoresis of the magnetic nanoparticles making the fluorescent superparamagnetic stream appear brighter. At the lowest flow rate of 2 µL/min, the magnetoconvective force is dominant and carries along the dye molecules until they reach the upper wall. If the flow rate increases, the hydrodynamic force outweighs the magnetoconvective force. Dye molecules, following the flow field, mostly travel in the direction of the pressure-driven main flow. On the other hand, superparamagnetic nanoparticles travel towards the interface due to the positive magnetophoresis effect. The ferrofluid becomes more diluted at y* > 0 as the flow approaches the outlet, and, consequently, the superparamagnetic stream becomes brighter. The effect is shown as subplots in Figure 2.

**Figure 3:**
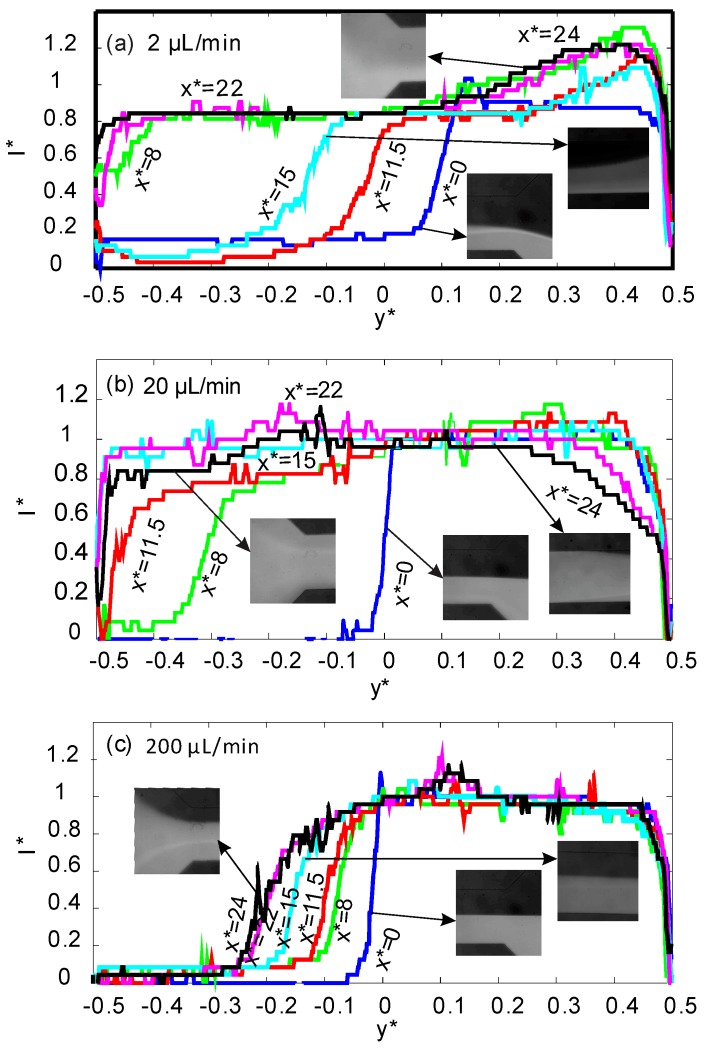
Normalized intensity across the normalized channel width y* for six positions of x* along the mixing channel with a ferrofluid concentration of 20% vol. (x* = 0: blue, x* = 8: green, x* = 11.5: red, x* = 15: cyan, x* = 22: purple, x* = 24: black) and three different flow rates: (**a**) 2 µL/min; (**b**) 20 µL/min; (**c**) 200 µL/min.

Figure 3 shows the relative intensity profiles for the higher concentration of 20% vol. At the lowest flow rate of 2 µL/min the residence time of the streams was longer, and the positive magnetophoresis effect on superparamagnetic nanoparticles was significant. Despite the fact that good mixing is achieved at the outlet, concentration distribution is not uniform: The ferrofluid is more diluted next to y* = 0.5 and more concentrated next to y* = −0.5. More homogeneous mixing was achieved at the higher flow rate of 20 µL/min. The high flow rate and the high magnetic susceptibility mismatch between the two streams allow for fast mixing. At the highest flow rate of 200 µL/min, as a result of the stronger hydrodynamic force in the flow direction, the superparamagnetic stream cannot reach the upper wall. Compared to the lower ferrofluid concentration of ϕ = 2% vol., the interface of ϕ = 20% vol. is closer to the upper wall (y* = −0.5). The images corresponding to the position of x* = 11.5 in Figure 3 demonstrate the secondary flow on the permanent magnets. At a lower flow rate of 2 µL/min (Figure 3a) the magnetic stream reaches the upper wall, and nanoparticle agglomeration occurs. At a flow rate of 20 µL/min, nanoparticle agglomeration disappears due to the higher hydrodynamic force. For the higher flow rate of 200 µL/min, secondary flow is not strong enough to reach the upper wall.

### 3.2. Mixing Efficiency, Probability Distribution, and Qualitative Analysis

The homogeneity of the mixed fluid indicates good mixing. Therefore, the distribution of the intensity values of an image can be used for evaluating the degree of mixing. By normalizing the pixel number of each intensity value by the total number of the pixels in the evaluated region, the probability values can be obtained [38]:

(2) $$P\left(c^{*}\right)=P\left(I^{*}\right)=P\left(I\right)=\frac{N\left(I\right)}{\sum_{I_{\min}}^{I_{\max}}N\left(I\right)}$$

We used a customised MATLAB (MathWorks) code to evaluate the probability distribution from the grayscale intensity images. If two intensity peaks appear on the probability graph (probability distribution function versus normalized intensity or concentration), then it means no mixing. In contrast, the existence of a single intensity peak indicates full mixing. Furthermore, the standard deviation can be normalized by the mean concentration to obtain the mixing efficiency:

(3) $$\eta_{\mathrm{mixing}}=1-\sqrt{\frac{1}{N}\overset{N}{\underset{i=1}{\sum }}{\left(\frac{I_{i}-\bar{I}}{\bar{I}}\right)}^{2}}$$

where $\eta_{\mathrm{mixing}}$ is the mixing efficiency, $I_{i}$ is the intensity value at a given position (pixel), and $\bar{I}$ is the average of intensity values of the region of interest. The mixing efficiency varies from 0 (no mixing) to 1 (full mixing).

Effective magnetic permeability of diluted ferrofluid emulsions could be estimated by the Maxwell-Garnett formula [39]:

(4) $$\mu_{ef}=\mu_{w}+3\mu_{w}\frac{\varphi \frac{\mu_{p}-\mu_{w}}{\mu_{p}+2\mu_{w}}}{1-\varphi \frac{\mu_{p}-\mu_{w}}{\mu_{p}+2\mu_{w}}}$$

where $\mu_{w}$ is the magnetic susceptibility of water, $\mu_{p}$ is the magnetic susceptibility of iron oxide particles, and $\mu_{ef}$ is the effective magnetic susceptibility of diluted ferrofluid.

**Figure 4:**
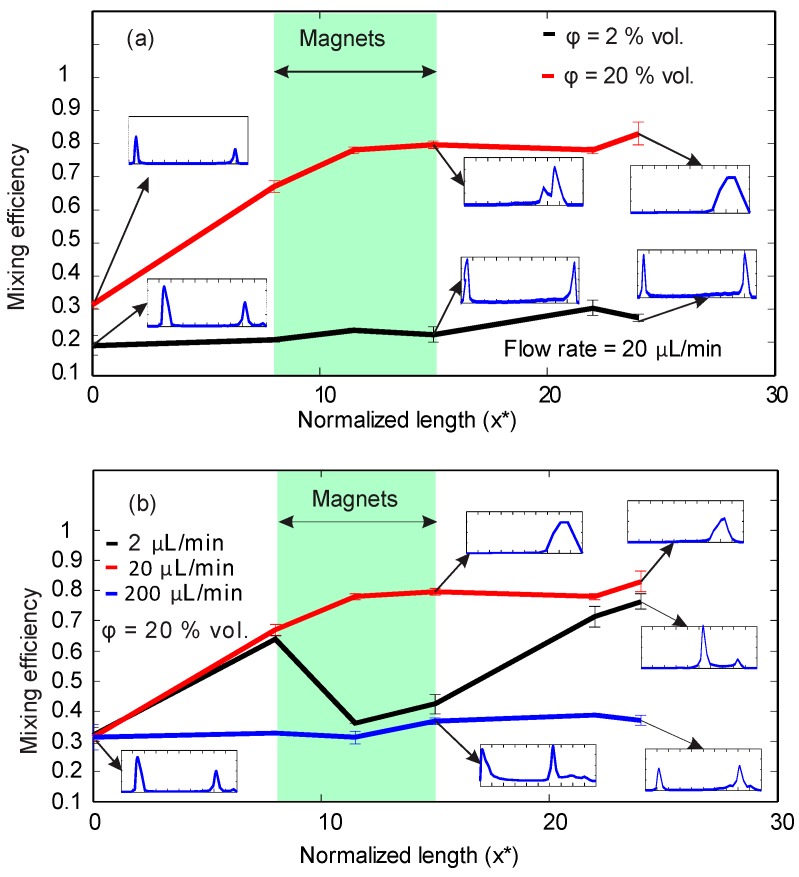
Mixing efficiency along the normalized channel length x* with probability distribution graphs of selected points: (**a**) Constant flow rate of 20 µL/min and ferrofluid concentrations of 2 and 20% vol.; (**b**) Constant concentration of 20% vol. and three flow rates of 2, 20, and 200 µL/min.

The images from experiments were taken at the six positions along the microchannel as mentioned above. The customized MATLAB code evaluated the mixing efficiency and probability distribution graphs for each position. Figure 4a shows the results of the analysis for concentrations of ϕ = 2% vol. and ϕ = 20% vol. at a constant flow rate of 20 µL/min. The results show the change of mixing efficiency from the inlet to the outlet. At a lower concentration of ϕ = 2% vol., the mixing efficiency remains around a low value of 0.2. The probability function also shows agreement with the mixing efficiency, suggesting no mixing occurred along the microchannel. With a higher concentration of ϕ = 20% vol., the mixing efficiency increases from about 0.3 at the inlet to around 0.8 at the outlet, indicating good mixing. The larger mismatch in magnetic susceptibility between the two streams and the high concentration of superparamagnetic nanoparticles created a strong secondary flow in the direction of the higher magnetic field. The magnetoconvective secondary flow causes a significant rise of mixing efficiency when the flow passes the permanent magnet region. The probability distribution graph also shows good mixing at the outlet for a higher concentration.

Figure 4b illustrates the effect of the flow rates (2, 20, 200 µL/min) at a constant concentration of 20%. For the lowest flow rate of 2 µL/min, a major drop of mixing efficiency can be observed at the location next to the magnets. The reason for this sudden decrease is the agglomeration of magnetic nanoparticles. The pile of accumulated nanoparticles creates a dark area with lower intensity. However, the mixing efficiency at the outlet of the device reaches a value of more than 0.7. The curve for the flow rate of 20 µL/min and a concentration of ϕ = 20% vol. was already discussed; a higher mixing efficiency was achieved as agglomeration problems do not exist. No mixing occurs at the higher flow rate of 200 µL/min due to the high hydrodynamic force which eliminates the magnetoconvection effect. Figure 4a,b indicate that best mixing result was obtained at the concentration of ϕ = 20% vol. and a flow rate of 20 µL/min.

### 3.3. Effect of Flow Rate and the Optimum Operating Conditions

Figure 4 indicates that a lower concentration of ϕ = 2% vol. does not create a strong enough magnetoconvective secondary flow for mixing. On the other hand, there must be an optimum flow rate for the highest mixing efficiency while at the same time avoiding nanoparticle accumulation. The mixing efficiency for the low flow rate of 2 μL/min shows a sudden decrease in the region between x* = 8 to 15 because of nanoparticle agglomeration next to the magnets. The pile of accumulated nanoparticles is dark and has a low intensity close to zero, Figure 3a. At this flow rate, the secondary flow is strong enough to fully mix the two streams. After x* = 15, where there is no agglomeration next to the upper wall, mixing efficiency rises to a higher value. We carried out mixing experiments for a wide range of flow rates, from 2 µL/min to 300 µL/min at the constant concentration of ϕ = 20% vol., to examine the effect of flow rate on mixing efficiency and to determine the optimum range for flow rate.

**Figure 5:**
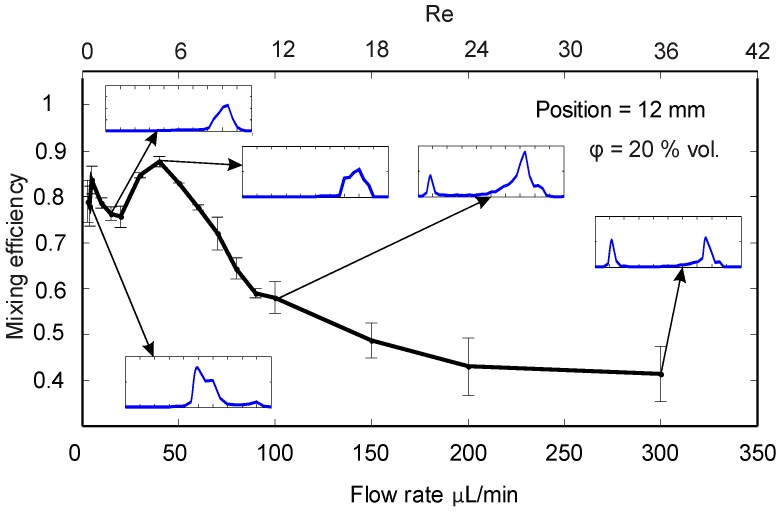
The effect of flow rate on mixing efficiency for a constant ferrofluid concentration of 20% at the outlet. The probability distribution graphs are indicated for the selected points.

Figure 5 shows the mixing efficiency values at the outlet versus flow rate. The probability graphs and mixing efficiency data show that low flow rates between 2 and 5 µL/min provide good mixing. The reason is the longer residence time of the superparamagnetic stream in the microchannel allowing for a better distribution of dye molecules across the channel width. Despite the high mixing efficiency, agglomeration of magnetic nanoparticles exists in that range of flow rates.

As the flow rate increases up to 20 µL/min, the mixing efficiency drops. At these medium flow rates, a concentrated substream of ferrofluid occurred near the upper wall (y* = −0.5). This sub-stream next to the upper wall is negligible at lower flow rates, and almost disappears at higher flow rates. At a flow rate of 45 µL/min, the device achieves the most homogenous mixing. At this flow rate, mixing was not affected by the accumulation of the nanoparticles or the concentrated stream next to the upper wall. As the flow rate increases beyond 45 µL/min, the high hydrodynamic force in the flow direction causes a drop in mixing efficiency. Although the probability graphs show one single peak for a wide range of flow rates, this tool was unable to identify a specific point for the optimum flow rate. Therefore, mixing index is a better indicator of efficient mixing for our device.

## 4. Conclusions

We proposed and investigated a simple micro-mixer based on magnetofluidic transport phenomena. The device takes advantage of a non-uniform magnetic field provided by permanent magnets. The magnetic susceptibility difference between a non-magnetic stream and a superparamagnetic stream leads to a magnetoconvective secondary flow toward a magnetic field maximum. The competition between this secondary flow and the pressure-driven hydrodynamic flow determines the extent of mixing. The superparamagnetic stream was diluted ferrofluid mixed with fluorescent dye. By analysing the fluorescent image, mixing was evaluated both qualitatively and quantitatively. Comparing the two concentrations of 2% vol. and 20% vol. indicates that the higher concentration delivers more efficient mixing. The effect of flow rate on mixing efficiency was also studied. An optimum flow rate range was determined to achieve efficient mixing and at the same time to avoid agglomeration of magnetic nanoparticles. The flow rate of 45 µL/min and the concentration of 20% vol. resulted in a mixing efficiency of 88% in our device. Considering the low cost, simplicity, and ability to achieve rapid mixing in a short microchannel, our micromixer has the potential to be implemented in lab-on-a-chip devices for chemical and biological studies. However, it is worth mentioning that the commercial ferrofluid used in this study contains surfactants, which contaminates fluids under mixing. Surfactants are hard to remove after the mixing, which changes the properties of the fluids under mixing. In addition, the effects of ferrofluid on chemical reactions is unknown. More work with customized ferrofluids is essential to discover the effect of surfactants on mixing efficiency and chemical reactions. Furthermore, studies to identify biocompatible ferrofluids are also needed for biological studies.
